# Differential association between metabolic syndrome and coronary artery disease evaluated with cardiac computed tomography according to the presence of diabetes in a symptomatic Korean population

**DOI:** 10.1186/1471-2261-14-105

**Published:** 2014-08-20

**Authors:** Ki-Bum Won, Hyuk-Jae Chang, Jimin Sung, Sanghoon Shin, In-Jeong Cho, Chi-Young Shim, Geu-Ru Hong, Young Jin Kim, Byung-Wook Choi, Namsik Chung

**Affiliations:** 1 Division of Cardiology, Yonsei Cardiovascular Center, Yonsei University College of Medicine, Seoul, Republic of Korea; 2 Division of Cardiology, St. Luke’s International Hospital, Tokyo, Japan; 3 Graduate School of Health and Welfare CHA University, Seongnam, Republic of Korea; 4 Division of Radiology, Yonsei Cardiovascular Center, Yonsei University College of Medicine, Seoul, Republic of Korea; 5 Severance Biomedical Science Institute, Seoul, Republic of Korea; 6 Yonsei Cardiovascular Center, Yonsei University College of Medicine, 50 Yonsei-ro, Seodaemun-gu, Seoul 120-752 Republic of Korea

**Keywords:** Metabolic syndrome, Diabetes, Coronary artery disease, Coronary computed tomographic angiography

## Abstract

**Background:**

Metabolic syndrome (MetS) is associated with increased risks of diabetes and coronary artery disease (CAD). Despite the controversial inclusion of established diabetes in MetS, the association between MetS and CAD according to diabetes status has not been elucidated in the Asian population.

**Methods:**

We evaluated the association between MetS and CAD using the parameters including any plaque, obstructive plaque, and coronary artery calcium score (CACS) >100 according to diabetes status in 2,869 symptomatic Korean subjects who underwent cardiac computed tomographic angiography.

**Results:**

The prevalence of MetS was significantly higher in the diabetic subjects than in the non-diabetic subjects (69% vs. 34%, P <0.001). The incidence of any plaque (64% vs. 43%, P <0.001), obstructive plaque (26% vs. 13%, P = 0.006), and CACS >100 (23% vs. 12%, P = 0.012) was significantly higher in diabetic subjects than in non-diabetic subjects. Among the MetS components, decreased high-density lipoprotein level was significantly associated with any plaque (odds ratio [OR] 1.35), obstructive plaque (OR 1.55), and CACS >100 (OR 1.57) in the non-diabetic subjects (P <0.01, respectively). However, none of the MetS components were associated with all the parameters in the diabetic subjects. Multivariate regression analysis revealed that MetS and the number of MetS components (MetSN) were independently associated with any plaque (MetS: OR 1.55, P <0.001; MetSN: OR 1.22, P <0.001), obstructive plaque (MetS: OR 1.52, P = 0.003; MetSN: OR 1.25, P <0.001), and CACS >100 (MetS: OR 1.46, P = 0.015; MetSN: OR 1.21, P = 0.004) only in the non-diabetic subjects, respectively.

**Conclusions:**

MetS was independently associated with the presence and severity of CAD only in the non-diabetic subjects among the symptomatic Korean population.

## Background

Metabolic syndrome (MetS) is a clustering condition of several cardiovascular (CV) risk factors, and it represents insulin resistance as a major characteristic [1]. MetS is associated with the development of coronary artery disease (CAD) and increased risk of CV morbidity and mortality [2]. MetS also increases the risk of diabetes development because its components represent major risk factors for impaired glucose metabolism [3]. Accordingly, MetS has been promoted as a means for identifying the risk of diabetes development in clinical practice. Recently, it has been strongly recommended that conditions with established diabetes should be excluded from the definition of MS [4]. However, data supporting this recommendation are limited, especially regarding coronary atherosclerosis.

A previous study reported that the prevalence of coronary heart disease (CHD) was significantly higher in diabetic subjects with MetS than in those without MetS [2]. However, this study defined CHD with only self-reported myocardial infarction or a positive response to the angina pectoris section of the Rose Questionnaire without an imaging approach for evaluating coronary atherosclerosis [5]. Accordingly, this study might underestimate the prevalence of CHD. In addition, this study was performed only in a Western (U.S) population, including African Americans and Mexican Americans.

Diabetes is a chronic, complex, and progressive illness that requires persistent medical care to prevent major CV complications. Diabetes significantly influences the development of CAD, and most diabetic subjects have MetS, which represents a major phenotype of insulin resistance. Although deterioration of insulin secretion and aggravation of insulin resistance are two major defects in the pathogenesis of diabetes [6,7], the clinical features of type 2 diabetes cases in Asia are explicitly different from those of type 2 diabetes cases in other parts of the world [8]. Several recent studies have reported that impaired insulin secretion was more prominent than insulin resistance, even in the status of impaired glucose tolerance, in Asian subjects [9,10]. Considering that MetS represents insulin resistance as a major characteristic, the impact of MetS on coronary atherosclerosis in diabetic subjects may be different in the Asian population compared with the Western population. However, the association between MetS, individual MetS components, and coronary atherosclerosis according to the presence of diabetes is not elucidated in an Asian population.

Coronary computed tomographic angiography (cCTA) was recently introduced as a novel noninvasive imaging approach for evaluating coronary atherosclerosis and for predicting CAD [11,12]. cCTA has high diagnostic accuracy in detecting CAD [13,14], and the coronary artery calcium score (CACS) is known as a good marker of CAD, representing the degree of atheromatous plaque burden [15,16]. In this study, we aim to evaluate the association between MetS, individual MetS components, and coronary atherosclerosis according to the presence of diabetes in symptomatic Korean subjects who underwent cardiac computed tomographic angiography.

## Methods

### Study population

This was a cross-sectional study analyzing single-center data collected from 3,159 consecutive symptomatic South Korean subjects who underwent cCTA evaluation with 64-slice multidetector computed tomography (MDCT) at Yonsei Cardiovascular Hospital between January 2005 and April 2009. All participants were referred for evaluation of CAD who had at least one of the symptoms, including typical angina, atypical angina, dyspnea, and excessive fatigue, but were not patients with acute coronary syndrome who required emergent coronary intervention or surgery. Subjects who were younger than 30 years (n = 26), or had an estimated modification of diet in renal disease (MDRD) glomerular filtration rate (GFR) <60 mL/min/1.73m^2^ (n = 264) were excluded from the present study according to the study protocol. As a result, 2,869 participants were finally included. All patients provided written informed consent, and ethical approval was obtained from the Institutional Review Board of Severance Hospital, Yonsei University Health System.

### MDCT protocol

Subjects with an initial heart rate ≥65 beats/min before MDCT examination received a single oral dose of 50 mg metoprolol tartrate (Betaloc, Yuhan, Seoul, South Korea) 1 h before CT examination unless beta-adrenergic blocking agents were contraindicated owing to overt heart failure, atrioventricular conduction abnormalities, and bronchial asthma. In patients with atrial fibrillation, patients with a mean heart rate >100 beats/min received beta-adrenergic blocking agents orally 1 h before cCTA. If the mean heart rate remained >100 beats/min at the time of scanning, we withdraw the scan. A contrast-enhanced volume data set was acquired with retrospective electrocardiogram gating without using tube current modulation to allow reconstructions during all phases of the cardiac cycle. In subjects with a mild allergy to the contrast material such as drug eruption or urticaria, we used a prophylactic IV steroid. However, we did not allow a scan for the patients with severe allergic reactions such as shock or laryngeal edema.

Imaging was performed for all the subjects using a 64-slice CT scanner (Sensation 64; Siemens Medical System, Forchheim, Germany). All CT examinations were performed during breath holding in inspiration. Initially, a non-enhanced prospective electrocardiogram-gated scan to evaluate CACS was performed with the following parameters: rotation time of 330 ms, slice collimation of 0.6 mm, slice width of 3.0 mm, tube voltage of 100–120 kV, tube current of 50 mA, and table feed/scan of 18 mm. Thereafter, cCTA was performed using retrospective electrocardiographic gating with the following scan parameters: rotation time of 330 ms, slice collimation of 64 × 0.6 mm, tube voltage of 100–120 kV, tube current of 400–800 mA (depending on patient size), table feed/scan of 3.8 mm, and pitch factor of 0.2. ECG-based tube current modulation was applied to 65% of the R–R interval. A real-time bolus-tracking technique was applied to trigger scan initiation. The total estimated average radiation dose for the multislice CT protocol was 8.8 ± 1.6 mSv. Contrast enhancement was achieved with 60 mL iopamidol (370 mg/mL iodine, Iopamiro; Bracco, Milan, Italy) injected at 5 mL/s, followed by an injection of 30 mL of diluted contrast medium (saline-to-contrast agent ratio, 7:3) and then 30 mL saline at 5 mL/s, with a power injector (Envision CT; Medrad, Indianola, PA) via an antecubital vein. The estimated volume CT dose index (CTDIvol) was recorded for each patient. The product of CTDIvol and scanning length (dose–length product, mGy × cm) was calculated, and effective dose was estimated using a normalization factor for the adult chest (0.017 mSv × mGy^−1^ × cm^−1^). Image reconstruction was performed in the scanner workstation using commercially available software (Wizard, Siemens Medical Solutions). Axial images were reconstructed retrospectively at 65% of the R–R interval for each cardiac cycle. If artifacts were present, additional data sets were obtained for various points of the cardiac cycle, and the data set with the minimum artifact was selected for further analysis. The reconstructed image data sets were transferred to an off-line workstation (Aquar-ius Workstation, TeraRecon, Inc., San Mateo, CA). Each lesion identified was examined using maximum intensity projection and multiplanar reconstruction techniques on a short axis and along multiple longitudinal axes. Lesions were classified by the maximal stenosis of luminal diameter observed on any plane.

### Measurement of coronary parameters

All cCTA were evaluated by 2 experienced cardiac radiologists (Y.J.K. and B.W.C., who respectively had 6 and 9 years of experience in cardiac CT). In case of disagreement, a joint reading was performed to reach a consensus. This study primarily evaluated the presence of any plaque, obstructive plaque, and a CACS >100. Both any plaque and obstructive plaque were divided into 2 subtypes according to the presence of coronary calcification as follows: calcified or mixed plaque (CMP) and non-calcified plaque (NCP). CACS was measured with the scoring system using a previously described method [17]. Because the frequency of CACS >100 in the Asian population is known to be low compared with that in Caucasians, African-Americans, and Hispanics [18], and few subjects had a CACS >400 (5%) in the present study, consequently, we used CACS >100 as the parameter for estimating severe coronary calcification. Plaque was defined as structures >1 mm^2^ within and/or adjacent to the vessel lumen that were clearly distinguished from the lumen and surrounding pericardial tissue; obstructive plaque was defined as plaque with ≥50% luminal diameter stenosis. CAD was defined as the presence of any coronary plaque identified in cCTA (both non-obstructive and obstructive lesions, including non-calcified plaques).

### Measurement of biochemical and clinical parameters

Information on medical history of hypertension, diabetes, and smoking status were systematically collected. Height, weight, and blood pressure were measured during hospital visits. All blood samples were obtained after a minimum 8-h fast and analyzed for triglycerides, high-density lipoprotein (HDL) cholesterol, low-density lipoprotein (LDL) cholesterol, and glucose levels. Body mass index (BMI) was calculated as weight (kg) ÷ height (m^2^), and obesity was defined as a BMI ≥25 kg/m^2^. Kidney function was assessed based on the estimated GFR calculated using the formula validated in the Modification of Diet in Renal Disease study [19]. Current smoking history was considered present if the subject consistently smoked or smoked within 1 month before the study. MetS was defined as the presence of ≥3 of the following: (a) blood pressure ≥130 mmHg systolic or ≥85 mmHg diastolic, or on antihypertensive treatment; (b) HDL cholesterol level <40 mg/dL in men or <50 mg/dL in women; (c) fasting triglycerides level ≥150 mg/dL; (d) BMI ≥25 kg/m^2^; and (e) impaired fasting glucose, defined as a fasting glucose level ≥100 mg/dL, a referral diagnosis of diabetes, or diabetes treatment according to the National Cholesterol Education Program–Adult Treatment Panel III definition [1]. Diabetes was defined as a fasting glucose level ≥126 mg/dL, undergoing an antidiabetic treatment, or a referral diagnosis of diabetes.

### Statistical analysis

Clinical, biochemical, and coronary characteristics are described according to the presence of MetS and diabetes. Values are expressed as mean ± SD or n (%). Continuous variables were compared using the Student t-test, and categorical variables were compared using the χ^2^ test. The associations between the individual MetS components and the coronary parameters, namely plaque, obstructive plaque, and CACS >100, were analyzed in the subjects with and without diabetes after adjusting for confounding risk factors. Univariate and multivariate logistic regression analyses for identifying the association between MetS and coronary parameters were performed according to diabetes status. These analyses were also performed to identify the association between the increase in number of MetS components (MetSN) and coronary parameters according to diabetes status. The covariate-adjusted odds ratios (OR) and 95% confidence intervals (CI) for each coronary parameter were calculated. Statistical Package for the Social Sciences version 18 (SPSS Inc., Chicago, IL) was used for all the analyses. All the statistical tests were 2-tailed, and P <0.05 was considered significant.

## Results

### Clinical characteristics

**Figure 1:**
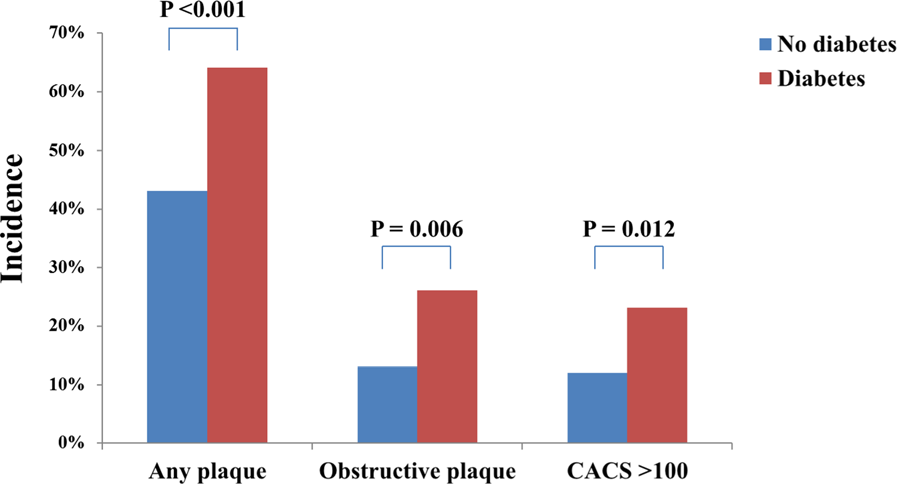
Comparison of the incidence of coronary parameters according to diabetes status.

**Figure 2:**
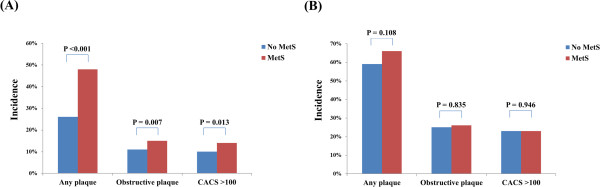
Comparison of the incidence of coronary parameters according to MetS status. (A) Non-diabetes and (B) diabetes.

The clinical characteristics of the 2,869 subjects (58 ± 9 years, 51% male) are listed in Table 1. Of the subjects, 2,308 were non-diabetic subjects (80%) and 561 were diabetic subjects (20%). The prevalence of MetS was significantly higher in the diabetic subjects than in the non-diabetic subjects (69% vs. 34%, P <0.001). The incidence of any plaque (64% vs. 43%, P <0.001), obstructive plaque (26% vs. 13%, P = 0.006), and CACS >100 (23% vs. 12%, P = 0.012) was also significantly higher in diabetic subjects than in non-diabetic subjects (Figure 1). The incidence of any plaque (48% vs. 39%, P <0.001), obstructive plaque (15% vs. 11%, P = 0.006), and CACS >100 (14% vs. 10%, P = 0.012), was significantly higher in the non-diabetic subjects with MetS than in those without MetS. However, the incidence of all these parameters was not significantly different in the diabetic subjects according to the presence of MetS (Figure 2).

**Table 1:** Baseline characteristics

**Characteristics**	**No diabetes**			**P**	**Type 2 diabetes**			**P**
	**Total**	**No MetS**	**MetS**		**Total**	**No MetS**	**MetS**	
	**(n = 2,308)**	**(n = 1,515)**	**(n = 793)**		**(n = 561)**	**(n = 176)**	**(n = 385)**	
Subjects								
Age (years)	57 ± 9	57 ± 9	57 ± 9	0.526	60 ± 9*	61 ± 9	60 ± 8	0.219
Men, n (%)	1,155 (50)	749 (49)	406 (51)	0.430	313 (56)^†^	105 (60)	208 (54)	0.234
Body mass index (kg/m^2^)	24.4 ± 2.8	23.6 ± 2.6	25.9 ± 2.5	<0.001	25.0 ± 3.0*	23.1 ± 2.3	25.9 ± 3.0	<0.001
Current smoking, n (%)	277 (12)	176 (12)	101 (13)	0.500	76 (14)	23 (14)	53 (14)	0.876
SBP (mmHg)	126 ± 15	124 ± 14	130 ± 15	<0.001	128 ± 15^†^	123 ± 13	130 ± 16	<0.001
DBP (mmHg)	79 ± 10	78 ± 9	81 ± 10	<0.001	78 ± 10	76 ± 9	79 ± 10	<0.001
Antihypertensive treatment, n (%)	1,098 (49)	628 (43)	470 (61)	<0.001	298 (54)^†^	61 (36)	237 (63)	<0.001
Total cholesterol (mg/dL)	188 ± 35	187 ± 34	190 ± 37	0.047	181 ± 37*	176 ± 36	183 ± 38	0.045
Triglyceride (mg/dL)	140 ± 91	115 ± 61	190 ± 114	<0.001	160 ± 102*	105 ± 50	185 ± 109	<0.001
HDL cholesterol (mg/dL)	51 ± 12	54 ± 12	45 ± 10	<0.001	48 ± 11*	54 ± 11	46 ± 10	<0.001
LDL cholesterol (mg/dL)	120 ± 33	120 ± 32	120 ± 34	0.850	114 ± 38*	115 ± 42	114 ± 36	0.670
Creatinine (mg/dL)	0.90 ± 0.16	0.90 ± 0.16	0.91 ± 0.16	0.182	0.91 ± 0.16	0.91 ± 0.17	0.90 ± 0.16	0.779
GFR (mL/min/1.73 m^2^)	82 ± 13	82 ± 13	82 ± 13	0.235	83 ± 14	83 ± 14	82 ± 14	0.513
FBS (mg/dL)	95 ± 10	93 ± 9	100 ± 11	<0.001	135 ± 43*	133 ± 42	135 ± 44	0.584
Duration of diabetes (years)	-	-	-	-	11 ± 7	12 ± 7	10 ± 7	0.094
Anti-diabetic treatment, n (%)	-	-	-	-	433 (77)	133 (76)	300 (78)	0.588
Coronary atherosclerosis parameters, n (%)								
Any plaque	981 (43)	597 (26)	384 (48)	<0.001	359 (64)*	104 (59)	255 (66)	0.108
CMP	797 (35)	480 (32)	317 (40)	<0.001	311 (55)*	96 (55)	215 (56)	0.784
NCP	184 (8)	117 (8)	67 (8)	0.571	48 (9)	8 (5)	40 (10)	0.022
Obstructive plaque	292 (13)	171 (11)	121 (15)	0.007	145 (26)*	44 (25)	101 (26)	0.835
Obstructive CMP	244 (11)	140 (9)	104 (13)	0.005	122 (22)*	39 (22)	83 (22)	0.912
Obstructive NCP	48 (2)	31 (2)	17 (2)	0.876	23 (4)^†^	5 (3)	18 (5)	0.366
CACS ≥ 100	264 (12)	155 (10)	109 (14)	0.013	129 (23)*	41 (23)	88 (23)	0.946

Data are expressed as n (%) or mean ± SD. CACS, coronary artery calcium score; CAD, coronary artery disease; CMP, calcified or mixed plaque; DBP, diastolic blood pressure; FBS, fasting blood sugar; GFR, glomerular filtration rate; HDL, high-density lipoprotein; LDL, low-density lipoprotein; MetS, metabolic syndrome; NCP, non-calcified plaque; SBP, systolic blood pressure. *P <0.001 vs. no diabetes. ^**†**^P <0.05 vs. no diabetes.

### Association individual MetS component and coronary parameters according to diabetes status

The association between the individual MetS components and the coronary parameters according to the presence of diabetes is presented in Table 2. After adjusting for age, sex, current smoking status, LDL, and GFR, obesity was significantly associated with any plaque (OR 1.27, 95% CI 1.05–1.55, P = 0.016). Increased blood pressure and increased triglycerides were significantly associated with any plaque (increased blood pressure: OR 1.25, 95% CI 1.01–1.55, P = 0.045; increased triglycerides: OR 1.37, 95% CI 1.12–1.68, P = 0.002) and obstructive plaque (increased blood pressure: OR 1.59, 95% CI 1.14–2.23, P = 0.006; increased triglycerides: OR 1.56, 95% CI 1.18–2.06, P = 0.002). Decreased HDL level was significantly associated with any plaque (OR 1.35, 95% CI 1.09–1.68, P = 0.007), obstructive plaque (OR 1.55, 95% CI 1.14–2.10, P = 0.005), and CACS >100 (OR 1.57, 95% CI 1.12–2.19, P = 0.008) in the non-diabetic subjects. However, no MetS components were significantly associated with all the coronary parameters in the diabetic subjects.

**Table 2:** Association between individual MetS component and coronary parameters according to diabetes status

**Characteristic**	**n**	**Any plaque**	**Obstructive plaque**	**CACS >100**
		**OR (95% CI)**	**OR (95% CI)**	**OR (95% CI)**
No diabetes	2308			
Obesity				
No	1383	1.00	1.00	1.00
Yes	925	1.27 (1.05–1.55) ^‡^	1.20 (0.91–1.59)	1.19 (0.88–1.62)
Increased blood pressure				
No	664	1.00	1.00	1.00
Yes	1644	1.25 (1.01–1.55)^‡^	1.59 (1.14–2.23)^†^	1.27 (0.89–1.81)
Increased triglycerides				
No	1522	1.00	1.00	1.00
Yes	786	1.37 (1.12–1.68)^†^	1.56 (1.18–2.06)^†^	1.18 (0.87–1.62)
Decreased HDL				
No	1616	1.00	1.00	1.00
Yes	692	1.35 (1.09–1.68)^†^	1.55 (1.14–2.10)^†^	1.57 (1.12–2.19)^†^
Increased fasting glucose				
No	1640	1.00	1.00	1.00
Yes	668	1.22 (0.99–1.50)	0.97 (0.72–1.31)	1.24 (0.90–1.69)
Type 2 diabetes	561			
Obesity				
No	288	1.00	1.00	1.00
Yes	273	1.27 (0.85–1.90)	0.86 (0.57–1.31)	1.12 (0.72–1.75)
Increased blood pressure				
No	140	1.00	1.00	1.00
Yes	421	1.03 (0.65–1.63)	0.76 (0.48–1.21)	1.11 (0.66–1.86)
Increased triglycerides				
No	315	1.00	1.00	1.00
Yes	246	1.34 (0.89–2.02)	1.00 (0.65–1.53)	0.93 (0.59–1.47)
Decreased HDL				
No	347	1.00	1.00	1.00
Yes	214	0.92 (0.60–1.40)	1.44 (0.93–2.24)	1.13 (0.70–1.82)

All models are adjusted for age, sex, current smoking, LDL, and GFR. CACS, coronary artery calcium score; CI, confidence intervals; GFR, glomerular filtration rate; HDL, high-density lipoprotein; LDL, low-density lipoprotein, MetS, metabolic syndrome; OR, odds ratios. *P <0.001; ^†^P <0.01; ^‡^P <0.05.

### Association between MetS, MetSN, and coronary parameters according to diabetes status

**Figure 3:**
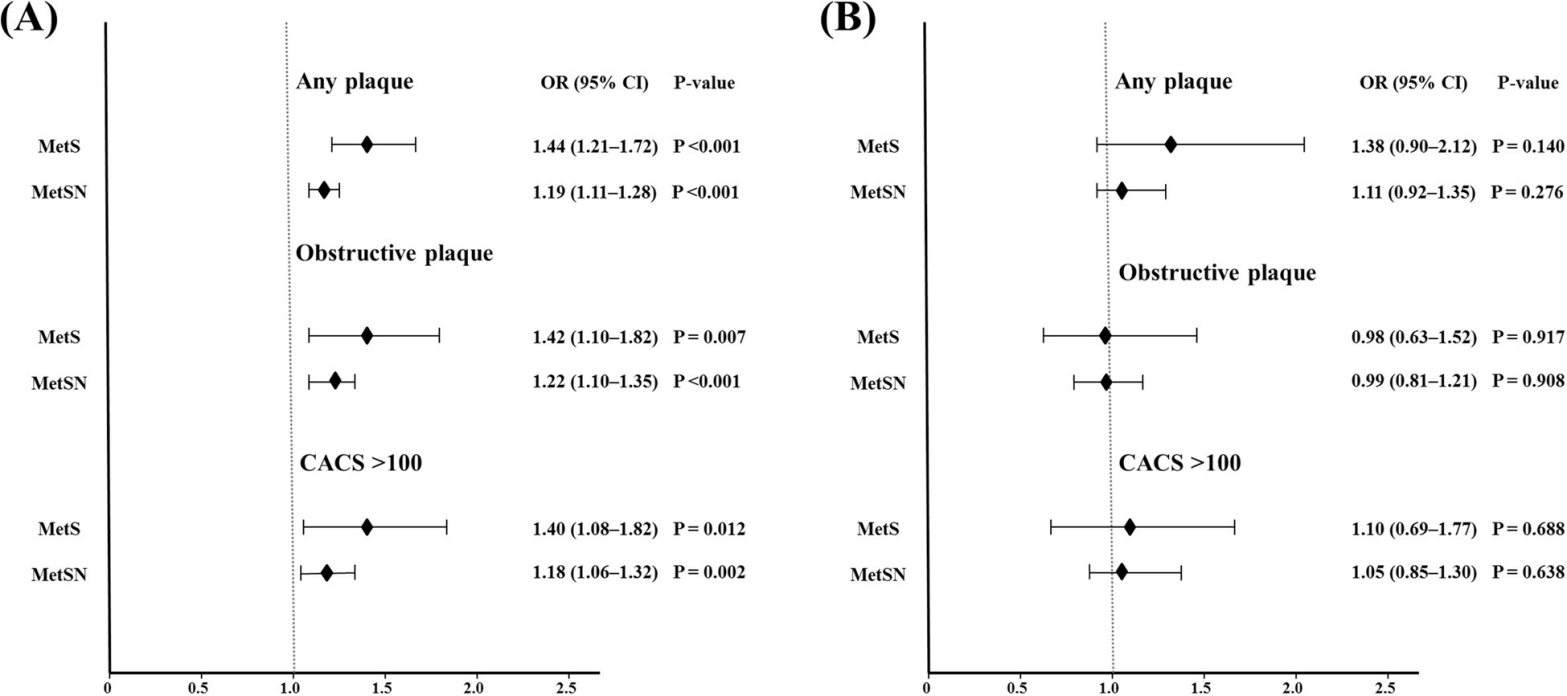
Impact of MetS and MetSN on the coronary parameters (multivariate analysis). (A) Non-diabetes and (B) diabetes.

The association between MetS, the MetSN, and the coronary parameters, including any plaque, obstructive plaque, and CACS >100 according to the presence of diabetes are presented in Table 3. The univariate logistic regression analysis revealed that MetS was significantly associated with increased risks of all coronary parameters in the non-diabetic subjects (any plaque: OR 1.44, 95% CI 1.21–1.72, P <0.001; obstructive plaque: OR 1.42, 95% CI 1.10–1.82, P = 0.007; CACS >100: OR 1.40, 95% CI 1.08–1.82, P = 0.012). However, MetS was not significantly associated with any of these parameters in the diabetic subjects. The MetSN was significantly associated with increased risks of all coronary parameters in the non-diabetic subjects (any plaque: OR 1.19, 95% CI 1.11–1.28, P <0.001; obstructive plaque: OR 1.22, 95% CI 1.10–1.35, P <0.001; CACS >100: OR 1.18, 95% CI 1.06–1.32, P = 0.002) but not in the diabetic subjects. After adjusting for age, sex, current smoking status, LDL, and GFR, MetS was significantly associated with increased risks of all the coronary parameters in the non-diabetic subjects (any plaque: OR 1.55, 95% CI 1.27–1.89, P <0.001; obstructive plaque: OR 1.52, 95% CI 1.15–2.01, P = 0.003; CACS >100: OR 1.46, 95% CI 1.08–1.99, P = 0.015). However, MetS was not significantly associated with any of these parameters in the diabetic subjects. The MetSN was significantly associated with increased risks of all the coronary parameters in the non-diabetic subjects (any plaque: OR 1.22, 95% CI 1.12–1.32, P <0.001; obstructive plaque: OR 1.25, 95% CI 1.11–1.41, P <0.001; CACS >100: OR 1.21, 95% CI 1.06–1.37, P = 0.004) but not in the diabetic subjects (Figure 3).

**Table 3:** Association between MetS, MetSN, and coronary parameters according to diabetes status

	**Any plaque**				**Obstructive plaque**				**CACS >100**			
	**Univariate**		**Multivariate**		**Univariate**		**Multivariate**		**Univariate**		**Multivariate**	
	OR	95% CI	OR	95% CI	OR	95% CI	OR	95% CI	OR	95% CI	OR	95% CI
No diabetes												
MetS	1.44	1.21–1.72*	1.55	1.27–1.89*	1.42	1.10–1.82^†^	1.52	1.15–2.01^†^	1.40	1.08–1.82^‡^	1.46	1.08–1.99^‡^
MetSN	1.19	1.11–1.28*	1.22	1.12–1.32*	1.22	1.10–1.35*	1.25	1.11–1.41*	1.18	1.06–1.32^†^	1.21	1.06–1.37^†^
Diabetes												
MetS	1.36	0.94–1.96	1.38	0.90–2.12	1.07	0.71–1.61	0.98	0.63–1.52	0.99	0.65–1.50	1.10	0.69–1.77
MetSN	1.06	0.89–1.25	1.11	0.92–1.35	1.01	0.84–1.21	0.99	0.81–1.21	0.94	0.78–1.14	1.05	0.85–1.30

CACS, coronary artery calcium score; CI, confidence intervals; LDL, low-density lipoprotein; MetS, metabolic syndrome; MetSN, number of metabolic syndrome components; OR, odd ratios.

Multivariate models are adjusted for age, sex, current smoking, LDL, and GFR. *P <0.001; ^†^P <0.01; ^‡^P <0.05

## Discussion

To the best of our knowledge, these are the first findings on the differential association between MetS and CAD according to diabetes status in a symptomatic Asian population. We identified that both MetS and MetSN were independently associated with the presence of plaque, obstructive plaque, and CACS >100 in the subjects without established diabetes. However, despite the significantly higher incidence of all the coronary parameters in the diabetic subjects than in the non-diabetic subjects, MetS, the individual MetS components, and the MetSN were not significantly associated with all these parameters in diabetic subjects.

The prevalence of MetS is rapidly increasing worldwide [20,21], and is affecting approximately 31% of adults in South Korea [22]. MetS and diabetes share many common characteristics, and 65–85% of diabetic patients have MetS [23,24]. Despite the similarities, MetS is considered as a pre-morbid condition rather than a clinical diagnosis and has been promoted as a means of identifying the risk of diabetes development [25]. However, most definitions of MetS simultaneously include diabetes in the diagnostic criteria as a component of impaired fasting glucose. The World Health Organization (WHO) strongly recommends that the conditions of established diabetes or CV disease should be excluded from MetS [4], but data supporting this recommendation are limited.

Previous studies have reported that the progression of atherosclerosis is independently associated with long-term hyperglycemia in patients with established diabetes [26,27]. Recently, Church et al. [28] reported that the presence of diabetes in the Aerobics Center Longitudinal Study was associated with a threefold greater mortality risk from CV disease and that MetS status did not modify this risk. Won et al. [29] reported that MetS was significantly associated with subclinical atherosclerosis, which is evaluated with brachial-ankle pulse wave velocity and carotid intima-media thickness and plaque, in conditions without diabetes and that a concurrent diagnosis of MetS might be of little value for predicting subclinical atherosclerosis in diabetic subjects. These results suggest the clinical usefulness of MetS for CV risk stratification only in subjects without established diabetes. Although MetS and diabetes are associated with an increased risk of major CV events, recent studies have suggested that people with these conditions face a wide range of increased risks of CV events and that coronary artery calcium is a good parameter for predicting CV events in people with MetS and diabetes [30-32]. Although these studies identified the strong influence of MetS and diabetes on coronary calcification, they did not evaluate the differential impact of MetS on coronary calcification according to diabetes status. Moreover, there is a paucity of data on the association between MetS and coronary plaque according to the presence of diabetes. The present study identified the different association between MetS and coronary atherosclerosis according to diabetes status. MetS was independently associated with any plaque, obstructive plaque, and CACS >100 only in the non-diabetic subjects after adjusting for confounding risk factors. In addition, although the incidence of these coronary parameters tended to increase according to the increase of MetSN in all participants (Additional file 1: Table S1), the MetSN was also independently associated with these coronary parameters only in the non-diabetic subjects. These results suggest that the progression of coronary atherosclerosis may be influenced by multiple metabolic risk factors in non-diabetic subjects but may be predominantly dependent on long-term hyperglycemia in subjects with established diabetes status [26,27].

The present study evaluated the association between the individual MetS component and coronary atherosclerosis according to the presence of diabetes. Among all the coronary atherosclerotic parameters, the identification of obstructive plaque might be the most important because this study was performed in symptomatic subjects. Although the measurement of CACS was not recommended in symptomatic subjects, the incidence of obstructive plaque was similar with that of CACS >100 in both the non-diabetic and diabetic subjects among the symptomatic Korean population. Interestingly, the components of increased blood pressure, increased triglycerides level, and decreased HDL level were independently associated with obstructive plaque in the non-diabetic subjects, but no MetS components were associated with obstructive plaque in the diabetic subjects. In the multivariate regression analysis for identifying the association between the individual MetS component and subtypes of obstructive plaque according to diabetes status, increased blood pressure, increased triglycerides level, and decreased HDL level were associated with obstructive CMP, not obstructive NCP, in the non-diabetic subjects. However, none of MetS components were associated with either obstructive CMP or obstructive NCP in the diabetic subjects (Additional file 2: Table S2). In the present study, we could not evaluate the association between impaired fasting glucose and obstructive plaque in the diabetic subjects because all of them had impaired fasting glucose. The component of impaired fasting glucose was unable to reflect differences in degree of hyperglycemic control, diabetes duration, and diabetes treatment in the diabetic subjects. Considering the significantly higher incidence of obstructive plaque in diabetic subjects than in non-diabetic subjects, these factors might be more closely related to obstructive plaque compared with the other MetS components in an established diabetic condition.

The present study has some limitations. First, we did not involve asymptomatic subjects in the present study. However, the application of cCTA for CV risk stratification in asymptomatic or low CV risk subjects has not been justified [33]. Second, we could not eliminate the possible effects of underlying medication on CAD because of the observational design of this study. Third, although we analyzed the association between MetS, individual MetS components, and plaque subtype, the incidence of NCP and obstructive NCP was too low to identify the impact of MetS and its individual component on these coronary parameters. Lastly, in the present study, we identified only the differential impact of MetS and its individual components on CAD, focusing on the presence of plaque, obstructive plaque, and coronary calcification, using MDCT according to diabetes status. Recent CT studies investigating patients with acute coronary syndrome have identified high-risk plaque features characteristic of culprit lesions, such as low plaque attenuation, positive remodeling, and spotty calcification [34,35]. Further large-scale investigations for identifying the impact of MetS and diabetes on plaque vulnerability may be necessary in subjects with suspected CAD.

Despite the several limitations of the present study, it was unique in that we identified the association between MetS and CAD according to the diabetic status of the subjects of Asian ethnicity only. Considering the clinical features of diabetic subjects in the Asian population might be explicitly different compared with those in a Western population, identification of the impact of MetS and its individual components on coronary atherosclerosis according to diabetes status in the Asian population might be important. The present study identified that MetS and the MetSN were independently associated with the presence and severity of CAD only in the non-diabetic subjects, despite the significant impact of diabetes on CAD in symptomatic Korean subjects. Moreover, this might provide clinical evidence related to the WHO recommendation that established diabetes should be excluded from MetS.

## Conclusions

In conclusion, MetS was independently associated with the presence and severity of CAD only in the non-diabetic subjects among the symptomatic Korean population. However, despite the significantly higher incidence of all the coronary parameters in the diabetic subjects than in the non-diabetic subjects, MetS, the individual MetS components, and the MetSN were not significantly associated with the presence and severity CAD in the diabetic subjects.

## Abbreviations

BMI: Body mass index (BMI); CACS: Coronary artery calcium score; CAD: Coronary artery disease; cCTA: Coronary computed tomographic angiography; CHD: Coronary heart disease; CI: Confidence intervals (CI); CMP: Calcified or mixed plaque; CV: Cardiovascular; GFR: Glomerular filtration rate; HDL: High-density lipoprotein; LDL: Low-density lipoprotein; MetS: Metabolic syndrome; MetSN: Number of MetS components; MDCT: Multidetector computed tomography; NCP: Non-calcified plaque; OR: Odds ratios

## Competing interests

The authors declare that they have no competing interests.

## Authors’ contributions

All authors have made substantial contributions. KW and HC conducted the design of the study. SS, IC, CS, and GH conducted all clinical measurements. YK and BC conducted all image measurements. KW and JS conducted the statistical analyses. NC reviewed, corrected and helped finalize the manuscript. All authors read and approved the final manuscript.

## Pre-publication history

The pre-publication history for this paper can be accessed here:

http://www.biomedcentral.com/1471-2261/14/105/prepub

## Supplementary Material

Additional file 1: Table S1 Comparison of incidence of coronary parameters according to MetSN. Click here for file

Additional file 2: Table S2 Impact of individual MetS component on subtypes of coronary plaque according to the diabetes status. Click here for file

## References

[B1] NCEP Executive summary of the third report of the national cholesterol education program (NCEP) expert panel on detection, evaluation, and treatment of high blood cholesterol in adults (adult treatment panel III JAMA 2001 285 2486 2497 10.1001/jama.285.19.2486 11368702

[B2] Alexander CM Landsman PB Teutsch SM Haffner SM NCEP-defined metabolic syndrome, diabetes, and prevalence of coronary heart disease among NHANES III participants age 50 years and older Diabetes 2003 52 1210 1214 10.2337/diabetes.52.5.1210 12716754

[B3] Haffner SM Valdez RA Hazuda HP Mitchell BD Morales PA Stern MP Prospective analysis of the insulin-resistance syndrome (syndrome X) Diabetes 1992 41 715 722 10.2337/diab.41.6.715 1587398

[B4] Simmons RK Alberti KG Gale EA Colagiuri S Tuomilehto J Qiao Q Ramachandran A Tajima N Brajkovich Mirchov I Ben-Nakhi A Reaven G Hama Sambo B Mendis S Roglic G The metabolic syndrome: useful concept or clinical tool? Report of a WHO expert consultation Diabetologia 2010 53 600 605 10.1007/s00125-009-1620-4 20012011

[B5] Rose GA Blackburn H Gillium RF Prineas RJ Cardiovascular Survey Methods 1982 2 Geneva: Switzerland, World Health Organization 4972212

[B6] Welsh M Mares J Oberg C Karlsson T Genetic factors of importance for beta-cell proliferation Diabetes Metab Rev 1993 9 25 36 10.1002/dmr.5610090104 8344120

[B7] DeFronzo RA Pathogenesis of type 2 diabetes mellitus Med clin North Am 2004 88 787 835 10.1016/j.mcna.2004.04.013 15308380

[B8] Yoon KH Lee JH Kim JW Cho JH Choi YH Ko SH Zimmet P Son HY Epidemic obesity and type 2 diabetes in Asia Lancet 2006 368 1681 1688 10.1016/S0140-6736(06)69703-1 17098087

[B9] Shin CS Lee HK Koh CS Kim YI Shin YS Yoo KY Paik HY Park YS Yang BG Risk factors for the development of NIDDM in Yonchon County, Korea Diabetes Care 1997 20 1842 1846 10.2337/diacare.20.12.1842 9405904

[B10] Kim DJ Lee MS Kim KW Lee MK Insulin secretory dysfunction and insulin resistance in the pathogenesis of korean type 2 diabetes mellitus Metabolism 2001 50 590 593 10.1053/meta.2001.22558 11319722

[B11] Budoff MJ Dowe D Jollis JG Gitter M Sutherland J Halamert E Scherer M Bellinger R Martin A Benton R Delago A Min JK Diagnostic performance of 64-multidetector row coronary computed tomographic angiography for evaluation of coronary artery stenosis in individuals without known coronary artery disease: results from the prospective multicenter ACCURACY (assessment by coronary computed tomographic angiography of individuals undergoing invasive coronary angiography) trial J Am Coll Cardiol 2008 52 1724 1732 10.1016/j.jacc.2008.07.031 19007693

[B12] Miller JM Rochitte CE Dewey M Arbab-Zadeh A Niinuma H Gottlieb I Paul N Clouse ME Shapiro EP Hoe J Lardo AC Bush DE de Roos A Cox C Brinker J Lima JA Diagnostic performance of coronary angiography by 64-row CT N Engl J Med 2008 359 2324 2336 10.1056/NEJMoa0806576 19038879

[B13] Hoffmann MH Shi H Schmitz BL Schmid FT Lieberknecht M Schulze R Ludwig B Kroschel U Jahnke N Haerer W Brambs HJ Aschoff AJ Noninvasive coronary angiography with multislice computed tomography JAMA 2005 293 2471 2478 10.1001/jama.293.20.2471 15914747

[B14] Cho I Min HS Chun EJ Park SK Choi Y Blumenthal RS Rivera JJ Nasir K Kim YJ Sohn DW Oh BH Park YB Chang HJ Coronary atherosclerosis detected by coronary CT angiography in asymptomatic subjects with early chronic kidney disease Atherosclerosis 2010 208 406 411 10.1016/j.atherosclerosis.2009.08.040 19781704

[B15] Budoff MJ Shaw LJ Liu ST Weinstein SR Mosler TP Tseng PH Flores FR Callister TQ Raggi P Berman DS Long-term prognosis associated with coronary calcification: observations from a registry of 25,253 patients J Am Coll Cardiol 2007 49 1860 1870 10.1016/j.jacc.2006.10.079 17481445

[B16] Detrano R Guerci AD Carr JJ Bild DE Burke G Folsom AR Liu K Shea S Szklo M Bluemke DA O'Leary DH Tracy R Watson K Wong ND Kronmal RA Coronary calcium as a predictor of coronary events in four racial or ethnic groups N Engl J Med 2008 358 1336 1345 10.1056/NEJMoa072100 18367736

[B17] Agatston AS Janowitz WR Hildner FJ Zusmer NR Viamonte M Jr Detrano R Quantification of coronary artery calcium using ultrafast computed tomography J Am Coll Cardiol 1990 15 827 832 10.1016/0735-1097(90)90282-T 2407762

[B18] Nasir K Shaw LJ Liu ST Weinstein SR Mosler TR Flores PR Flores FR Raggi P Berman DS Blumenthal RS Budoff MJ Ethnic differences in the prognostic value of coronary artery calcification for all-cause mortality J Am Coll Cardiol 2007 50 953 960 10.1016/j.jacc.2007.03.066 17765122

[B19] Levey AS Coresh J Balk E Kausz AT Levin A Steffes MW Hogg RJ Perrone RD Lau J Eknoyan G National Kidney Foundation practice guidelines for chronic kidney disease: evaluation, classification, and stratification Ann Intern Med 2003 139 137 147 10.7326/0003-4819-139-2-200307150-00013 12859163

[B20] Mozumdar A Liguori G Persistent increase of prevalence of metabolic syndrome among U.S. adults: NHANES III to NHANES 1999–2006 Diabetes Care 2011 34 216 219 10.2337/dc10-0879 20889854 PMC3005489

[B21] Nestel P Lyu R Low LP Sheu WH Nitiyanant W Saito I Tan CE Metabolic syndrome: recent prevalence in East and Southeast Asian populations Asia Pac J Clin Nutr 2007 16 362 367 17468095

[B22] Lim S Shin H Song JH Kwak SH Kang SM Won YJ Choi SH Cho SI Park KS Lee HK Jang HC Koh KK Increasing prevalence of metabolic syndrome in Korea: the Korean National Health and Nutrition Examination Survey for 1998–2007 Diabetes Care 2011 34 1323 1328 10.2337/dc10-2109 21505206 PMC3114326

[B23] Koehler C Ott P Benke I Hanefeld M Comparison of the prevalence of the metabolic syndrome by WHO, AHA/NHLBI, and IDF definitions in a German population with type 2 diabetes: the diabetes in Germany (DIG) study Horm Metab Res 2007 39 632 635 10.1055/s-2007-985816 17846969

[B24] Tong PC Kong AP So WY Yang X Ho CS Ma RC Ozaki R Chow CC Lam CW Chan JC Cockram CS The usefulness of the international diabetes federation and the national cholesterol education Program’s adult treatment panel III definitions of the metabolic syndrome in predicting coronary heart disease in subjects with type 2 diabetes Diabetes Care 2007 30 1206 1211 10.2337/dc06-1484 17259472

[B25] Gupta AK Prieto-Merino D Dahlöf B Sever PS Poulter NR Metabolic syndrome, impaired fasting glucose and obesity, as predictors of incident diabetes in 14120 hypertensive patients of ASCOT-BPLA: comparison of their relative predictability using a novel approach Diabet Med 2011 28 941 947 10.1111/j.1464-5491.2011.03330.x 21749444

[B26] Sander D Schulze-Horn C Bickel H Gnahn H Bartels E Conrad B Combined effects of hemoglobin A1c and C-reactive protein on the progression of subclinical carotid atherosclerosis: the INVADE study Stroke 2006 37 351 357 10.1161/01.STR.0000199034.26345.bc 16373634

[B27] Larsen JR Brekke M Bergengen L Sandvik L Arnesen H Hanssen KF Dahl-Jorgensen K Mean HbA1c over 18 years predicts carotid intima media thickness in women with type 1 diabetes Diabetologia 2005 48 776 779 10.1007/s00125-005-1700-z 15759107

[B28] Church TS Thompson AM Katzmarzyk PT Sui X Johannsen N Earnest CP Blair SN Metabolic syndrome and diabetes, alone and in combination, as predictors of cardiovascular disease mortality among men Diabetes Care 2009 32 1289 1294 10.2337/dc08-1871 19366967 PMC2699717

[B29] Won KB Chang HJ Kim HC Jeon K Lee H Shin S Cho IJ Park SH Lee SH Jang Y Differential impact of metabolic syndrome on subclinical atherosclerosis according to the presence of diabetes Cardiovasc Diabetol 2013 12 41 10.1186/1475-2840-12-41 23452437 PMC3599532

[B30] Bulugahapitiya U Siyambalapitiya S Sithole J Idris I Is diabetes a coronary risk equivalent? systematic review and metaanalysis Diabet Med 2009 26 142 148 10.1111/j.1464-5491.2008.02640.x 19236616

[B31] Wong ND Nelson JC Granston T Bertoni AG Blumenthal RS Carr JJ Guerci A Jacobs DR Jr Kronmal R Liu K Saad M Selvin E Tracy R Detrano R Metabolic syndrome, diabetes, and incidence and progression of coronary calcium: the Multiethnic Study of Atherosclerosis study JACC Cardiovasc Imaging 2012 5 358 366 10.1016/j.jcmg.2011.12.015 22498324 PMC3327555

[B32] Malik S Budoff MJ Katz R Blumenthal RS Bertoni AG Nasir K Szklo M Barr RG Wong ND Impact of subclinical atherosclerosis on cardiovascular disease events in individuals with metabolic syndrome and diabetes: the multi-ethnic study of atherosclerosis Diabetes Care 2011 34 2285 2290 10.2337/dc11-0816 21844289 PMC3177707

[B33] Cho I Chang HJ Sung JM Pencina MJ Lin FY Dunning AM Achenbach S Al-Mallah M Berman DS Budoff MJ Callister TQ Chow BJ Delago A Hadamitzky M Hausleiter J Maffei E Cademartiri F Kaufmann P Shaw LJ Raff GL Chinnaiyan KM Villines TC Cheng V Nasir K Gomez M Min JK Coronary computed tomographic angiography and risk of all-cause mortality and nonfatal myocardial infarction in subjects without chest pain syndrome from the CONFIRM Registry (coronary CT angiography evaluation for clinical outcomes: an international multicenter registry) Circulation 2012 126 304 313 10.1161/CIRCULATIONAHA.111.081380 22685117

[B34] Hoffmann U Moselewski F Nieman K Jang IK Ferencik M Rahman AM Cury RC Abbara S Joneidi-Jafari H Achenbach S Brady TJ Noninvasive assessment of plaque morphology and composition in culprit and stable lesions in acute coronary syndrome and stable lesions in stable angina by multidetector computed tomography J Am Coll Cardiol 2006 47 1655 1662 10.1016/j.jacc.2006.01.041 16631006

[B35] Motoyama S Sarai M Harigaya H Anno H Inoue K Hara T Naruse H Ishii J Hishida H Wong ND Virmani R Kondo T Ozaki Y Narula J Computed tomographic angiography characteristics of atherosclerotic plaques subsequently resulting in acute coronary syndrome J Am Coll Cardiol 2009 54 49 57 10.1016/j.jacc.2009.02.068 19555840

