# Supplementary material for: Differential association between metabolic syndrome and coronary artery disease evaluated with cardiac computed tomography according to the presence of diabetes in a symptomatic Korean population
Source: BMC Cardiovasc Disord. 2014 Aug 20;14:105. doi: 10.1186/1471-2261-14-105 (PMC4236521; doi:10.1186/1471-2261-14-105)
Supplement: Additional file 1: Table S1 — Comparison of incidence of coronary parameters according to MetSN. [file 1471-2261-14-105-S1.doc]

**Table S1** Comparison of incidence of coronary parameters according to MetSN

|  | No MetS component  (n = 200) | 1 MetS component  (n = 630) | 2 MetS components  (n = 861) | 3 MetS components  (n = 728) | 4 MetS components  (n = 356) | 5 MetS components  (n = 94) | P |
| --- | --- | --- | --- | --- | --- | --- | --- |
| Plaque, n (%) | 68 (34) | 252 (40) | 381 (44) | 379 (52) | 205 (58) | 55 (59) | <0.001 |
| Obstructive plaque, n (%) | 14 (7) | 77 (12) | 124 (14) | 124 (17) | 76 (21) | 22 (23) | <0.001 |
| CACS >100, n (%) | 16 (8) | 64 (10) | 116 (14) | 120 (17) | 59 (17) | 18 (19) | <0.001 |

CACS, coronary artery calcium score; MetS, metabolic syndrome; MetSN, number of metabolic syndrome components.
