# Supplementary material for: Differential association between metabolic syndrome and coronary artery disease evaluated with cardiac computed tomography according to the presence of diabetes in a symptomatic Korean population
Source: BMC Cardiovasc Disord. 2014 Aug 20;14:105. doi: 10.1186/1471-2261-14-105 (PMC4236521; doi:10.1186/1471-2261-14-105)
Supplement: Additional file 2: Table S2 — Impact of individual MetS component on subtypes of coronary plaque according to the diabetes status. [file 1471-2261-14-105-S2.doc]

**Table S2** Impact of individual MetS component on subtypes of coronary plaque according to the diabetes status

| Characteristic | n | Any CMP  OR (95% CI) | Any NCP  OR (95% CI) | Obstructive CMP  OR (95% CI) | Obstructive NCP  OR (95% CI) |
| --- | --- | --- | --- | --- | --- |
| No diabetes | 2308 |  |  |  |  |
| Obesity  No  Yes | 1383  925 | 1.00  1.43 (1.16‒1.76)^†^ | 1.00  0.82 (0.59‒1.16) | 1.00  1.28 (0.94‒1.73) | 1.00  0.94 (0.50‒1.74) |
| Increased blood pressure  No  Yes | 664  1644 | 1.00  1.32 (1.05‒1.66)^‡^ | 1.00  0.92 (0.64‒1.31) | 1.00  1.80 (1.23‒2.63)^†^ | 1.00  0.96 (0.50‒1.85) |
| Increased triglycerides  No  Yes | 1522  786 | 1.00  1.40 (1.13‒1.73)^†^ | 1.00  1.07 (0.76‒1.51) | 1.00  1.54 (1.13‒2.08)^†^ | 1.00  1.56 (0.85‒2.87) |
| Decreased HDL  No  Yes | 1616  692 | 1.00  1.31 (1.04‒1.65)^‡^ | 1.00  1.21 (0.84‒1.74) | 1.00  1.57 (1.12‒2.19)^†^ | 1.00  1.38 (0.72‒2.66) |
| Increased fasting glucose  No  Yes | 1640  668 | 1.00  1.26 (1.01‒1.57)^‡^ | 1.00  0.99 (0.69‒1.43) | 1.00  1.03 (0.75‒1.41) | 1.00  0.78 (0.39‒1.56) |
| Type 2 diabetes | 561 |  |  |  |  |
| Obesity  No  Yes | 288  273 | 1.00  1.10 (0.74‒1.61) | 1.00  1.52 (0.78‒2.95) | 1.00  0.81 (0.52‒1.26) | 1.00  1.23 (0.47‒3.21) |
| Increased blood pressure  No  Yes | 140  421 | 1.00  1.04 (0.67‒1.63) | 1.00  0.97 (0.46‒2.06) | 1.00  0.72 (0.44‒1.17) | 1.00  1.14 (0.37‒3.53) |
| Increased triglycerides  No  Yes | 315  246 | 1.00  1.00 (0.67‒1.48) | 1.00  2.34 (1.18‒4.63)^‡^ | 1.00  0.89 (0.56‒1.40) | 1.00  1.70 (0.64‒4.48) |
| Decreased HDL  No  Yes | 347  214 | 1.00  0.81 (0.54‒1.23) | 1.00  1.40 (0.71‒2.79) | 1.00  1.38 (0.87‒2.20) | 1.00  1.48 (0.55‒3.97) |

All models are adjusted for age, sex, current smoking, LDL, and GFR. BMI, body mass index; CA, coronary atherosclerosis; CACS, coronary artery calcium score; CAD, coronary artery disease; CI, confidence intervals; CMP, calcified or mixed plaque; GFR, glomerular filtration rate; HDL, high-density lipoprotein; LDL, low-density lipoprotein, MetS, metabolic syndrome; NCP, non-calcified plaque; OR, odds ratios. *P <0.001; ^†^P <0.01; ^‡^P <0.05.
